# Toll-like receptor expression in crypt epithelial cells, putative stem cells and intestinal myofibroblasts isolated from controls and patients with inflammatory bowel disease

**DOI:** 10.1111/cei.12381

**Published:** 2014-09-04

**Authors:** M Brown, K R Hughes, S Moossavi, A Robins, Y R Mahida

**Affiliations:** * Institute of Infection, Immunity and Inflammation, University of NottinghamUK; † Nottingham Digestive Diseases Centre, University of NottinghamUK; ‡ Division of Immunology, University of NottinghamUK

**Keywords:** Crohn's disease, ulcerative colitis

## Abstract

The aim of our studies was to investigate the expression of Toll-like receptor (TLR)-2 and TLR-4 (and in some studies TLR-5) in myofibroblasts and small and large intestinal crypt epithelial cells from control patients and those affected by Crohn's disease and ulcerative colitis. Isolated and disaggregated crypt epithelial cells and monolayers of myofibroblasts were used for studies by reverse transcription–polymerase chain reaction (RT–PCR), real-time RT–PCR, flow cytometry, immunocytochemistry and Western blot analysis. Compared to control cells, crypt epithelial cells isolated from active ulcerative colitis and Crohn's disease colonic mucosal samples showed significantly higher expression of TLR-2 and TLR-4 transcripts and protein (on the cell surface). There was also enhanced expression of TLR-4 in crypt cells from ileal Crohn's disease. Expression of TLR-2 and TLR-4 transcripts in crypt epithelial cells isolated from inflamed mucosa of distal ulcerative colitis did not differ significantly from such cells obtained from the normal proximal colon. Crypt epithelial cells with side population characteristics (putative stem cells) also expressed transcripts and protein for TLR-2, TLR-4 and TLR-5. Colonic myofibroblast expression of these TLRs was much weaker than in crypt epithelial cells. In conclusion, enhanced TLR-2 and TLR-4 expression by crypt epithelial cells in active inflammatory bowel disease likely reflects greater ability to respond to microbial products. Results from our studies using mucosal samples from patients with distal ulcerative colitis suggest that the enhanced expression of these TLRs could be constitutive. TLR-2, TLR-4 and TLR-5 expression by stem cells imply ability to respond to distinct bacterial products.

## Introduction

The inflammatory bowel diseases (IBD), ulcerative colitis and Crohn's disease, are a group of chronic conditions affecting the gastrointestinal tract characterized by a relapsing and remitting course. Although the pathogenesis of IBD remains to be fully understood, studies have implicated the epithelium, innate and adaptive immunity and resident (commensal) bacteria in disease pathogenesis 1,2.

The intestinal epithelium consists of a monolayer of subpopulations of cells of distinct phenotype and function, which are derived from stem cells located in crypts 3,4. There is increasing recognition of the importance of interactions between intestinal epithelial cells and commensal bacteria (and their products) in the maintenance of normal mucosal homeostasis 5. Changes in the nature of these interactions are also believed to be required for the development of chronic inflammatory disease of the intestine, as seen in IBD 1,2. In addition to providing a physical barrier to penetration by resident bacteria and their products, epithelial cells may also shape immune responses mediated by cells in the lamina propria. This may occur via specific receptors which recognize and respond to bacterial products. Toll-like receptors (TLRs) are the best-known sensors of microbial components 6, and act by regulating gene expression.

Studies in mice have shown that TLR-2, TLR-4 and TLR-5 control intestinal epithelial homeostasis and provide protection from injury, such as that mediated by dextran sodium sulphate and radiation 7–10. TLR-2 and TLR-4 sense Gram-positive and Gram-negative bacterial wall components (lipoteichoic acid and lipopolysaccharide, respectively) and TLR-5 binds monomeric flagellin of motile bacterial cell walls. Although agonists for these TLRs have been reported to provide protection against radiation injury 10,11, there are conflicting reports regarding their role in established murine models of IBD 12.

Human intestinal epithelial expression of TLR-2 and TLR-4 has been studied in tissue sections. In histologically normal colonic tissue the findings have been inconsistent, with epithelial expression reported to be confined to crypts 13,14, minimally detectable 15 or absent 16. In tissue affected by IBD, epithelial expression of these TLRs was reported to be absent 16 or abundant for TLR-4 15.

In patients with active IBD, changes in the epithelium are prominent and include loss of barrier function and loss of monolayer continuity (ulceration). During remission, epithelial repair and regeneration occurs via stem cell-derived progeny and processes such as restitution. Epithelial barrier function 17 and restitution 18 can be regulated by myofibroblasts that are located under the basement membrane. Myofibroblasts are also believed to be important regulators of intestinal stem cell function, via secretion of Wnt ligands 19 and bone morphogenetic protein (BMP) antagonists 20. Moreover, myofibroblasts are capable of responding to luminal bacterial products via expression of TLRs 21,22, but relative expression compared to epithelial cells is unknown.

The aims of our studies were to investigate the expression of TLR-2, TLR-4 and TLR-5 in human intestinal crypt epithelial cells and putative stem cells isolated from control tissue and that affected by IBD. Myofibroblast expression of the TLRs was also studied.

## Materials and methods

### Patients and samples

For isolation of crypt epithelial cells, mucosal samples were obtained from operation resection specimens. These included colonic tissue from 18 patients with ulcerative colitis [mean age 51·5, standard error of the mean (s.e.m.) 4·75 years], 11 patients with Crohn's colitis (48·9, s.e.m. = 4·10 years) and 11 patients (70·5, s.e.m. = 4·15 years) undergoing colonic resection for cancer (for control, histologically normal mucosal samples, > 5 cm from tumour). Control and inflamed ileal mucosal samples were obtained from seven patients (74·1, s.e.m. = 3·36 years) undergoing right hemicolectomy for cancer and seven patients (44·3, s.e.m. = 7·18 years) with ileal Crohn's disease.

Histological examination of mucosal samples from patients with inflammatory bowel disease showed mild to severe inflammation and they were on the following treatment at the time of intestinal resection: mesalazine (14), corticosteroids (eight), azathioprine/6-mercaptopurine (15), methotrexate (four), infliximab/adalimumab (eight), cyclosporin (one), metronidazole (one) (see Supporting information, Table S1). Specimens from patients who had received pre-operative chemotherapy or radiotherapy of any type or duration were excluded.

The above mucosal samples, which were surplus to clinical requirements, were used following informed consent from patients. This research was approved by the Nottingham Research Ethics Committee.

### Isolation and disaggregation of crypt epithelial cells

Intestinal crypts were isolated and disaggregated as described previously 23,24. In brief, after washing with calcium- and magnesium-free Hanks's balanced salt solution (HBSS), mucosal strips were incubated (for 30 min at 37°C, with shaking), on three occasions in 1 mM ethylenediaminete traacetic acid (EDTA) plus 0·05 mM dithiothreitol (DTT). Between the incubation steps, the mucosal strips were washed with HBSS. Released crypts were subsequently disaggregated using 0·25% pancreatin (Sigma, St Louis, MO, USA) and the cell suspensions were stored on ice prior to use in experiments.

### Myofibroblast isolation and co-culture with crypt epithelial cells

Primary colonic myofibroblasts were isolated and cultured as described previously 25. Briefly, mucosal samples denuded of epithelial cells (as described above), were cultured [at 37°C, in RPMI-1640 supplemented with 10% fetal calf serum (FCS)] to allow myofibroblasts to migrate out via basement membrane pores and to establish in culture. Established colonies of myofibroblasts were cultured in Dulbecco's modified Eagle's medium (DMEM) supplemented with 10% FCS, 1% non-essential amino acids (Gibco, Carlsbad, CA, USA), and 200 mM glutamine (Sigma). Following passage, the myofibroblasts were kept frozen prior to their use in experiments. Expression of α-smooth muscle actin and vimentin (by immunohistochemistry) confirmed the phenotype of the myofibroblasts.

For co-culture, myofibroblasts were grown to confluency on sterile glass coverslips before application of isolated and disaggregated crypt epithelial cells, as described previously 24. Following co-culture for 30 min, non-adherent cells were removed by washing before the coverslips were fixed in cold acetone for 1 min and stored at −20°C until required.

### Immunocytochemistry

Immunocytochemistry was undertaken as reported previously 24 using Vectastain ABC Universal kit (Vector Laboratories, Peterborough, UK). Cells on coverslips were thawed, hydrated and endogenous peroxidase activity was quenched in 0·3% H_2_O_2_ in methanol for 30 min. Coverslips were incubated with the following primary antibodies (for 1 h at room temperature or overnight at 4°C): anti-BerEP4 (Dako, Glostrup, Denmark), anti-α-smooth muscle actin (abcam, Cambridge, UK), anti-vimentin (abcam), anti-desmin (abcam), anti-TLR-2 (eBioscience, San Diego, CA, USA), anti-TLR-4 (eBioscience) or anti-TLR-5 antibody (Santa Cruz Biotechnology, Santa Cruz, CA, USA). Following incubation with biotinylated secondary antibody and avidin–biotin complex, peroxidase activity was developed with diaminobenzidene (DAB) solution as per the manufacturer's instructions.

### Flow cytometry and cell sorting

Isolated and disaggregated crypt epithelial cells were incubated (in the dark at 4°C for 1 h) with the following fluorophore-conjugated monoclonal antibodies: BerEP4-fluorescein isothiocyanate (FITC) (Dako), immunoglobulin (Ig)G2aκ isotype control-allophycocyanin (APC) (eBioscience), TLR-2-APC (eBioscience), TLR-4-APC (eBioscience) or CD45-AF488 (BioLegend, London, UK). Additional control included incubation in medium only (no primary antibody). Cells were subsequently washed, resuspended in 0·5% formaldehyde in phosphate-buffered saline (PBS) and stored at 4°C in the dark until analysis. A minimum of 20 000 total events per sample tube were collected for analysis on Moflo XDP (Beckman Coulter, High Wycombe, UK). Initial analysis was by forward- and side-scatter to exclude aggregates and non-viable cells. Side population cells were identified as reported previously 24,26.

Disaggregated crypt epithelial cells were used with and without prior incubation (at 37°C for 15 min) with either 50 μmol/l verapamil (Sigma-Aldrich, St Louis, MO, USA) or 10 μmol/l fumitremorgin C (Alexis Biochemicals, Exeter, UK). Hoechst 33342 (Sigma) was added to a final concentration of 2·5 μg/ml and the cells incubated (in the dark) for 30 min at 37°C, followed by 30 min at 4°C. Following centrifugation, the cells were resuspended in 0·5 ml of 2% fetal calf serum in HBSS with Ca/Mg and 10 mM HEPES, followed by incubation with normal mouse serum and fluorophore-conjugated monoclonal antibodies (above) for 1 h at 4°C. Following resuspension in medium at 4°C, the cells were analysed immediately on Moflo XDP (Beckman Coulter).

Viable and non-aggregated crypt cells were identified using forward- and side-scatter analysis and lack of cellular uptake of propidium iodide (PI), and analysed on a Beckman Coulter MoFlo cell sorter. Hoechst 33342 was excited at 405 nm and fluorescence emission measured using a 450/50 nm band-pass filter (‘Hoechst blue’) and a 620 nm long-pass filter (‘Hoechst red’).

Side population cells were demonstrated as those with low fluorescence in both the blue and red channels, which was ameliorated in cells pre-incubated with verapamil or fumitremorgin C, which block multi-drug resistance protein (mdr) or mdr-like mediated efflux of the Hoechst dye 26. Following measurement of the fluorescence signal in the relevant gated region, 5 × 10^3^ side population cells were sorted into Eppendorf tubes on ice and centrifuged, before total RNA was isolated for subsequent mRNA expression analysis, as described below. All flow cytometric data were analysed using Weasel version 3 software.

### RT–PCR (conventional and real-time)

Total RNA was extracted using Qiagen RNeasy Plus Mini Kit (Qiagen, Venlo, the Netherlands), as per the manufacturer's instructions for eukaryotic cellular RNA. Synthesis of complementary DNA (cDNA) from mRNA was undertaken using the Qiagen QuantiTect RT kit (Qiagen), according to the manufacturer's instructions.

The following primer pairs were used for conventional reverse transcription–polymerase chain reaction (RT–PCR): hypoxanthine–guanine phosphoribosyltransferase (HPRT) sense 5′-GAC CAG TCA ACA GGG GAC AT-3′; HPRT anti-sense 5′-CGA CCT TGA CCA TCT TTG GA-3′ [to give a 160 base pairs (bp) PCR product]; TLR-2 sense 5′-AGT TGA TGA CTC TAC CAG ATG-3′; TLR-2 anti-sense 5′-GTC AAT GAT CCA CTT GCC AG-3′ (599 bp PCR product); TLR-4 sense 5′-TGG ATA CGT TTC CTT ATA AG-3′; and TLR-4 anti-sense 5′-GAA ATG GAG GCA CCC CTT C-3′ (507 bp PCR product). Primers for TLR-5 were also used (QuantiTect Primer Assay primers; Qiagen), according to the manufacturer's instructions. Following RT–PCR, nucleic acid amplicons were separated by 1% agarose gel electrophoresis and visualized using ethidium bromide ultraviolet (UV)-transillumination. Amplicon specificity was confirmed by sequencing the PCR products.

For real-time RT–PCR reactions, the following primer pairs were used: HPRT sense 5′-GAC CAG TCA ACA GGG GAC AT-3′; HPRT anti-sense 5′-CGA CCT TGA CCA TCT TTG GA-3′; TLR-2 sense 5′-GGG TTG AAG CAC TGG ACA AT-3′; TLR-2 anti-sense 5′-CTG CCC TTG CAG ATA CCA TT-3′; TLR-4 sense 5′-CGG AGG CCA TTA TGC TAT GT-3′; and TLR-4 anti-sense 5′-TCC CTT CCT CCT TTT CCC TA-3′. Real-time RT–PCR studies used the SYBR green method (QuantiTect SYBR green kit; Qiagen), using Stratagene MX4000 real-time PCR cycler and MxPro Mx3000P version 4·01 software (Stratagene, La Jolla, CA, USA). The RT–PCR reactions (in triplicate) for colonic crypt epithelial cells were undertaken in one run and those for crypt cells from the small intestine in a separate run. Relative quantification of the transcripts of interest (in cells isolated from ulcerative colitis and Crohn's disease mucosal samples) was deduced by comparing the cycle threshold (Ct) value of each sample to the mean Ct value of the control group, normalizing to housekeeping gene HPRT, as reported previously 27. Data are presented as a ‘fold change’ in expression of transcript in each sample compared to mean expression in the control group.

### Western blot analysis

Disaggregated crypt epithelial cells (10^6^) or colonic myofibroblasts were washed in PBS and incubated in CelLytic M reagent (Sigma) supplemented with phosphatase inhibitor cocktail 2 (Sigma) and protease inhibitor cocktail (Sigma), according to the manufacturer's instructions. The lysate was centrifuged at 10 000 ***g*** and the protein-containing supernatant was stored at −80°C until required.

Aliquots of total protein, mixed in a 1:1 ratio with Laemmli buffer (Bio-Rad, Hercules, CA, USA), were separated by sodium dodecyl sulphate-polyacrylamide gel electrophoresis (SDS-PAGE) before transfer to a polyvinylidene difluoride (PVDF) membrane (GE Healthcare, Little Chalfont, UK).

The PVDF membrane was incubated (at 4°C) overnight with or without the following antibodies: anti-β-actin (Sigma), anti-TLR-2 (eBioscience) and anti-TLR-4 (abcam). Immunostaining was performed using a Vectastain ABC Universal kit (Vector Laboratories), according to the manufacturer's instructions.

### Statistical analyses

Normally distributed data were analysed using paired or unpaired Student's *t*-test, as appropriate. Non-normally distributed data were analysed using non-parametric tests, Kruskal–Wallis test and either a Wilcoxon signed-rank test or Mann–Whitney *U*-test. Categorical data were analysed using Fisher's exact test. Statistical analyses were undertaken using spss (version 19) and Graphpad Prism (version 5) statistical software packages. All statistical tests were two-tailed and those with *P*-values less than 0·05 (5%) were deemed statistically significant.

## Results

### Expression of TLR-2 and TLR-4 transcripts in colonic crypt epithelial cells

**Fig 1:**
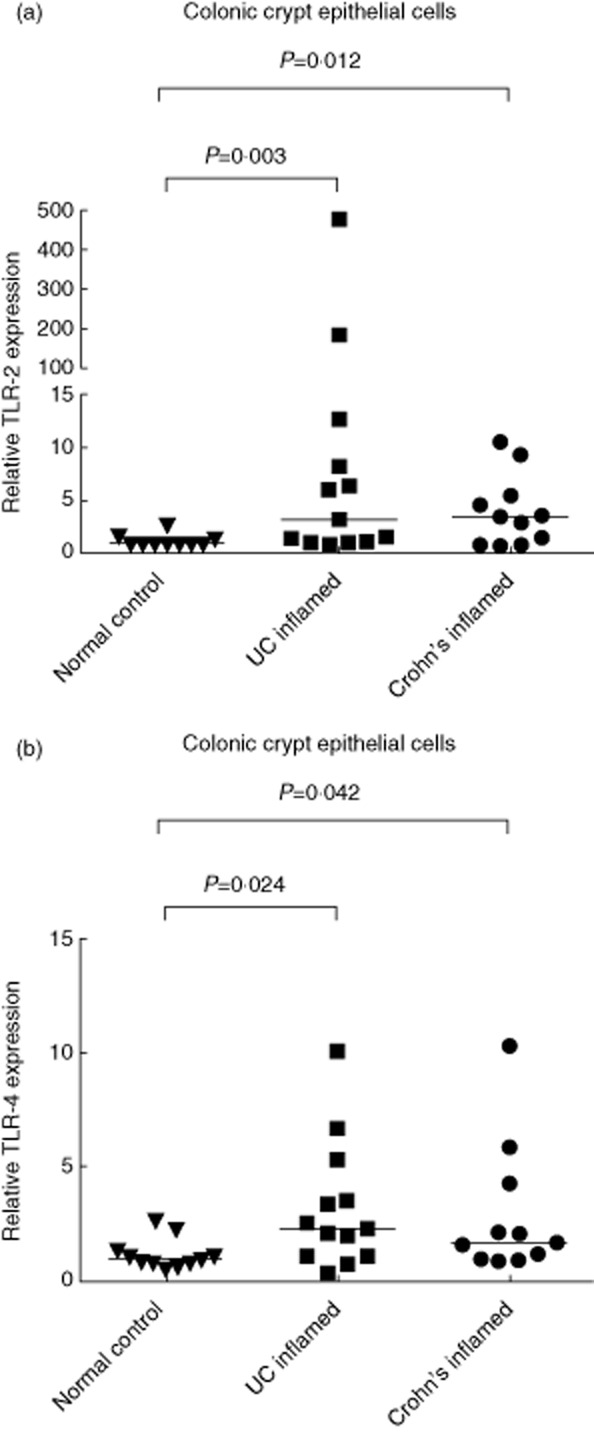
Relative quantitative expression of Toll-like receptor (TLR)-2 (a) and TLR-4 (b) mRNA transcripts in isolated and disaggregated colonic crypt epithelial cells obtained from histologically normal control mucosal samples (*n* = 11) and those affected by active ulcerative colitis (UC, *n* = 13) and Crohn's colitis (*n* = 11). Extracted RNA was used for real-time reverse transcription–polymerase chain reaction (RT–PCR) and data for UC and Crohn's colitis are presented as ‘fold change’ in expression of transcripts compared to mean expression in the control group in which the crypt epithelial cells were obtained from histologically normal colonic mucosal samples. Each data point represents mean mRNA expression of three samples per patient and the horizontal bars represent median expression.

Conventional RT–PCR studies using isolated and disaggregated crypt epithelial cells from normal control and IBD mucosal samples showed amplification products specific for TLR-2 and TLR-4 (see Supporting information, Figure S1). In studies using real-time RT–PCR, compared to normal control colonic mucosal samples, crypt epithelial cells isolated from inflamed ulcerative colitis (UC) and inflamed Crohn's colitis mucosal samples showed significantly increased expression of TLR-2 [median (range) fold increase (relative to mean, corrected to 1·0, of normal control cells): UC 3·18 (0·91–474·9), *P* = 0·003; Crohn's colitis 3·45 (0·75–10·51), *P* = 0·012; Fig. 1a] and TLR-4 [fold increase: UC 2·33 (0·40–10·07), *P* = 0·024; Crohn's colitis 1·71 (0·91–10·30), *P* = 0·042; Fig. 1b and Supporting information Table S2]. In the UC group, the two highest values of TLR-2 expression were from crypt cells isolated from severely inflamed specimens.

Compared to cells from normal control colonic mucosal samples, there was enhanced expression of TLR-4 mRNA in crypt cells isolated from histologically normal mucosal samples obtained from the right colon of five patients with left-sided UC [fold increase: 1·90 (1·63–5·75), *P* = 0·017]. Difference in the expression of TLR-2 mRNA transcripts between these two groups [UC fold increase: 1·36 (0·75–5·77), *P* = 0·254] did not reach statistical significance.

It is of interest that there were no statistically significant differences in expression of TLR-2 [median (range) fold increase (relative to mean, corrected to 1·0, of normal control cells): inflamed distal: 1·14 (0·89–8·93) *versus* 1·4 (0·23–8·65)] and TLR-4 [2·56 (0·4–3·54) and 1·9 (1·16–5·76)] mRNA between crypt cells isolated from inflamed (distal colon) and histologically normal proximal colon of the five patients with left-sided ulcerative colitis.

### Expression of TLR-2 and TLR-4 transcripts in ileal crypt epithelial cells

**Fig 2:**
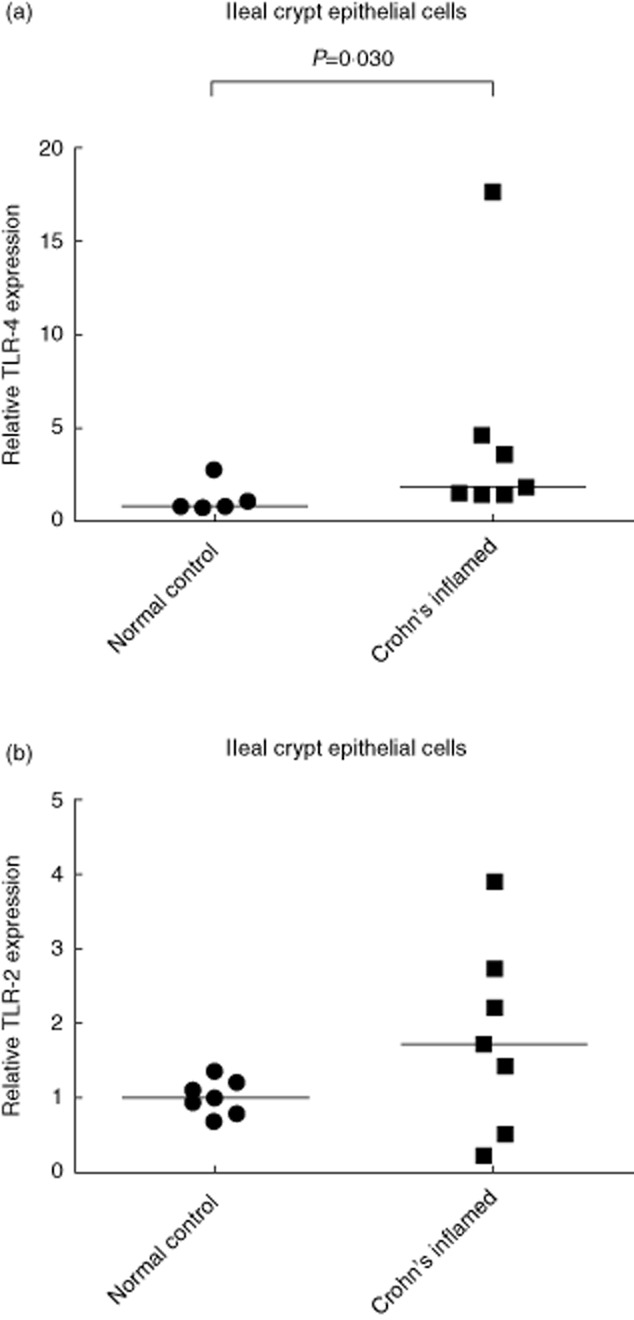
Relative quantitative expression of Toll-like receptor (TLR)-4 (a) and TLR-2 (b) mRNA transcripts in isolated and disaggregated ileal crypt epithelial cells obtained from mucosal samples affected by active Crohn's disease (*n* = 7) and histologically normal control ileal tissue (*n* = 7). Extracted RNA was used for real-time reverse transcription–polymerase chain reaction (RT–PCR) and data for Crohn's ileal crypt cells are presented as ‘fold change’ in expression of transcripts compared to mean expression in cells from the control group. Each data point represents mean mRNA expression of three samples per patient and the horizontal bars represent median expression.

There was significantly enhanced expression of TLR-4 transcripts in crypt cells isolated from inflamed ileal Crohn's disease mucosal samples, when compared to cells obtained from normal control ileal tissue [fold increase: 1·84 (1·39–17·69), *P* = 0·030; Fig. 2a]. Although some Crohn's ileal crypt cell samples showed high levels of TLR-2 mRNA expression, the difference between the two groups was not statistically significant [fold increase for Crohn's group: 1·72 (0·23–3·89); Fig. 2b and Supporting information, Table S2].

### Surface crypt epithelial expression of TLR-2 and TLR-4

**Fig 3:**
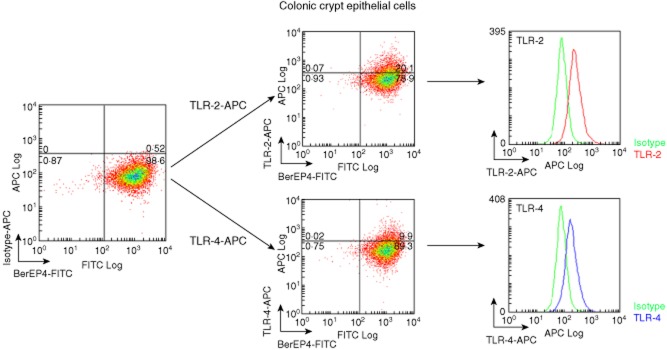
Toll-like receptor (TLR)-2 and TLR4 protein expression on the surface of isolated and disaggregated colonic crypt epithelial cells. The cells were incubated with anti-BerEP4-fluorescein isothiocyanate (FITC) antibody, followed by anti-TLR-2-allophycocyanin (APC), anti-TLR-4-APC or isotype control monoclonal antibodies. The cells were subsequently fixed and analysed by flow cytometry. TLR-2 (upper middle and right panels) and TLR-4 (lower middle and right panels) expression was determined in BerEP4-positive (gated) cells, which represent intestinal epithelial cells. The histograms show median fluorescent intensity of crypt epithelial cells labelled with anti-TLR-2 (red line, upper right panel) and anti-TLR-4 (blue line, lower right panel) monoclonal antibodies, or isotype control antibody (green line). The figure is representative of the experimental data summarized in Fig. 4, using crypt epithelial cells isolated from histologically normal control and inflamed (ulcerative colitis and Crohn's colitis) colonic mucosal samples.

Studies by flow cytometry showed that most of the isolated and disaggregated crypt cells obtained from normal control colon [mean 90·7 (s.e.m. ± 2·1)%], ulcerative colitis [92·4 (s.e.m. ± 3·0)%] and colonic Crohn's disease [90·0 (s.e.m. ± 2·1)%] tissue expressed the epithelial cell-specific marker Ber-EP4 (Fig. 3). Small populations of non-epithelial cells (identified by expression of CD45) were also present in disaggregated crypt cell preparations from normal control colon [1·5 (s.e.m. ± 0·4)%], ulcerative colitis [2·4 (s.e.m. ± 1·3)%] and colonic Crohn's disease [2·5 (s.e.m. ± 0·7)%] tissue. Immunocytochemical staining of cytospin preparations using anti-Ber-EP4 and anti-CD45 antibodies confirmed these findings (data not shown).

**Fig 4:**
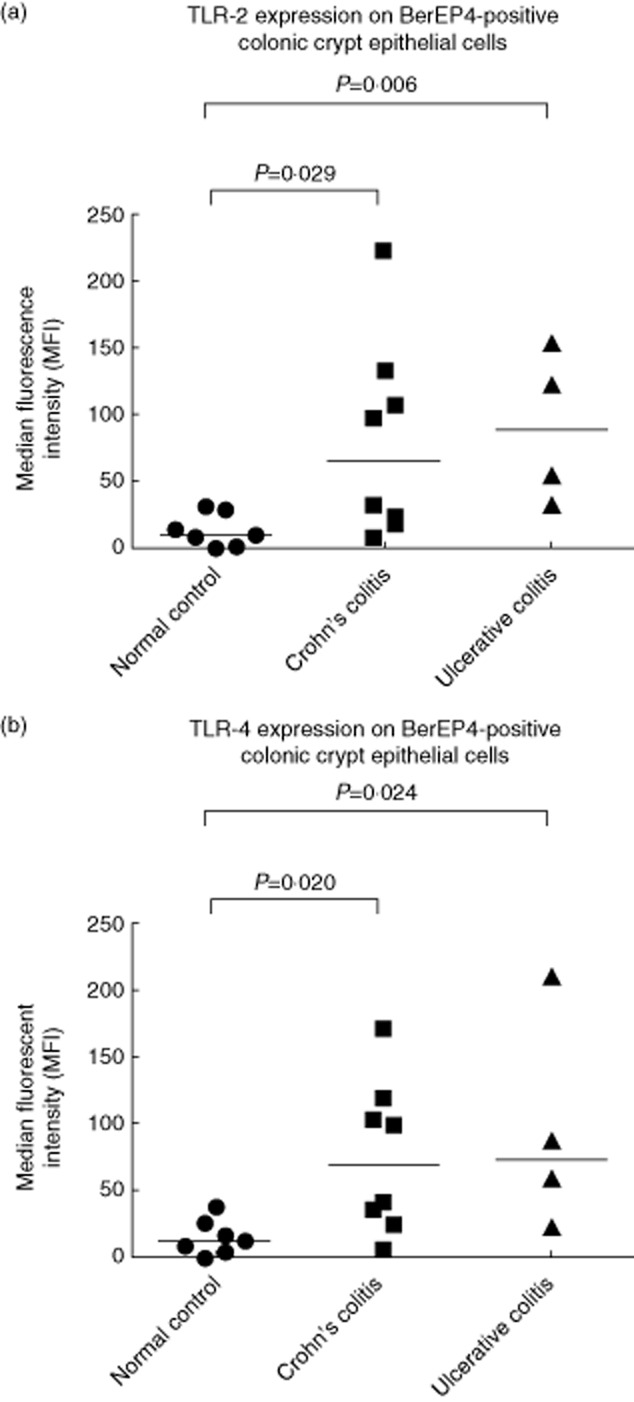
Quantitative surface Toll-like receptor (TLR)-2 and TLR-4 protein expression by colonic crypt epithelial cells. Isolated and disaggregated crypt epithelial cells were obtained from mucosal samples affected by active Crohn's colitis (*n* = 8), active ulcerative colitis (*n* = 4) or from histologically normal control colonic tissue (*n* = 7). The cells were labelled with anti-BerEP4-fluorescein isothiocyanate (FITC) antibody and either anti-TLR2-allophycocyanin (APC), anti-TLR-4-APC or isotype control monoclonal antibodies and analysed by flow cytometry. Surface TLR-2 (a) and TLR-4 (b) protein expression was assessed in BerEP4-positive (gated) epithelial cells. Each data point represents the difference in median fluorescent intensity (MFI) between the primary and isotype control antibodies. Horizontal bars represent median values.

Compared to cells isolated from normal control colonic mucosal samples [median (range) fluorescence intensity: TLR-2, 10·1 (0·50–31·40); TLR-4, 12·10 (4·90–37·4)], BerEP4-positive crypt epithelial cells isolated from inflamed UC and Crohn's colitis mucosal samples demonstrated significantly greater expression of surface TLR2 [median fluorescence intensity: UC 89·10 (33·40–153·90), *P* = 0·006; Crohn's colitis 65·80 (8·50–222·70), *P* = 0·029; Fig. 4a and Supporting information, Table S3] and TLR-4 [UC 72·95 (21·90–210·10), *P* = 0·024; Crohn's colitis 69·70 (6·40–170·90), *P* = 0·020; Fig. 4b and Supporting information, Table S3]. The presence of TLR-2 and TLR-4 transcripts in sorted BerEP4-positive cells was confirmed by RT–PCR (not shown).

### TLR expression by putative stem cells

**Fig 5:**
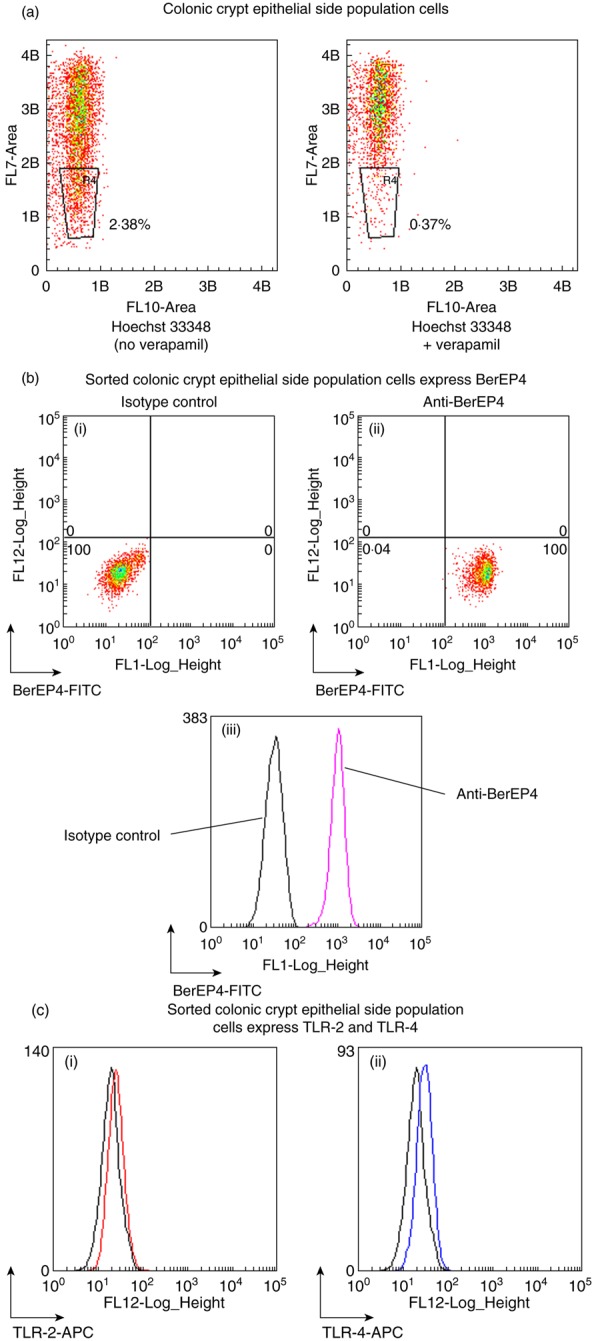
Characterization of side population cells (putative stem cells). (a) Identification of cells with side population characteristics in isolated and disaggregated crypt epithelial cells. Crypt epithelial cells isolated from histologically normal colonic mucosal samples were incubated with Hoechst 33348 either alone (left panel) or following exposure to verapamil (right panel). The small population of cells with low fluorescence in both the red (FL10) and blue (FL7) channels (region R4; 2·38% in left panel) was identified, and most of the cells in this region showed side population characteristics, as illustrated by amelioration of low florescence (in both channels) in the presence of verapamil (right panel). (b) Side population cells express BerEP4. Sorted side population cells were labelled with isotype control (i) or anti-BerEP4-fluorescein isothiocyanate (FITC)-conjugated (ii) monoclonal antibodies. The histogram (iii) shows median fluorescent intensity of side population cells labelled with anti-BerEP4 monoclonal antibody. (c) Side population cells express Toll-like receptor (TLR)-2 and TLR-4. Sorted and labelled with anti-TLR-2-allophycocyanin (APC), anti-TLR-4-APC or isotype control monoclonal antibodies. The histograms show median fluorescent intensity of side population cells labelled with anti-TLR-2 (i; red), anti-TLR-4 (ii; blue) or isotype control (i, ii; black) monoclonal antibodies. The figures are representative of crypt epithelial cells isolated from 12 [(a) and (b)] and six [(c)] operation resection specimens.

**Fig 6:**
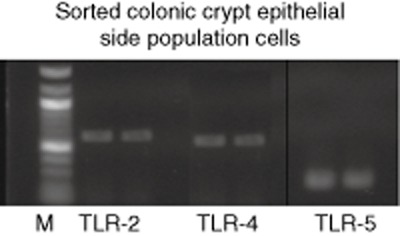
Side population cells (putative stem cells) express Toll-like receptor (TLR)-2, TLR-4 and TLR-5 mRNA transcripts. Side population cells in isolated and disaggregated colonic crypt epithelial cells were identified (as described in Fig. 5a) and sorted for extraction of RNA, for subsequent reverse transcription–polymerase chain reaction (RT–PCR) using primer pairs specific for TLR-2, TLR-4 and TLR-5. Lane M represents the 100 base pair size ladder. The figure is representative of crypt epithelial cells isolated from three operation resection specimens.

**Fig 7:**
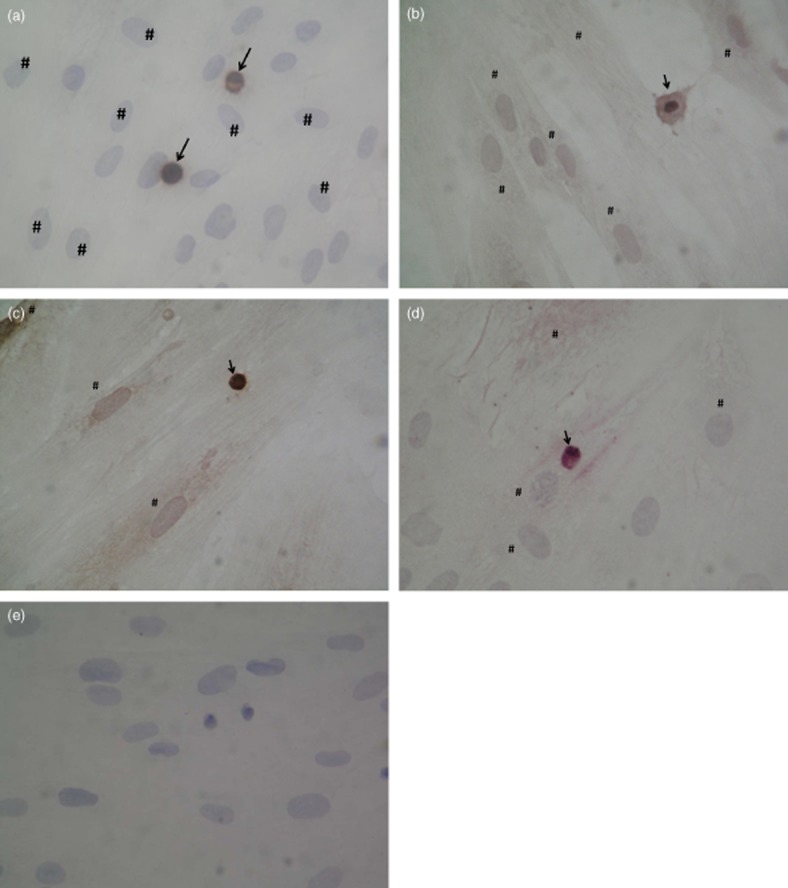
Expression of BerEP4 (a), Toll-like receptor (TLR)-2 (b), TLR-4 (c) and TLR-5 (d) protein in co-cultures of myofibroblasts and adherent crypt epithelial cells [(e) is negative control]. Isolated and disaggregated colonic crypt epithelial cells were cultured (at 37°C for 30 min) on monolayers of primary human colonic myofibroblasts. After washing, the myofibroblasts and adherent crypt epithelial cells (which are enriched for side population cells) were fixed and used for immunocytochemistry using relevant specific monoclonal antibodies in (a–d), or control buffer (e). Immunolabelled crypt epithelial cells (arrowed) are seen adherent to the much larger underlying myofibroblasts. Myofibroblasts (majority indicated by #) are negative for epithelial cell-specific BerEP4 (hence only their nuclei are seen) and weakly positive for TLR-2, TLR-4 and TLR-5. Each figure is representative of co-cultures using cells isolated from > 5 resection specimens.

Side population cells present in isolated and disaggregated crypt cell preparations from normal control colon were characterized by flow cytometry (Fig. 5a), as described previously 24. Sorted side population cells were labelled by anti-BerEP4 (Fig. 5b), anti-TLR-2 and anti-TLR-4 (Fig. 5c) antibodies. When studied by RT–PCR, sorted side population cells also expressed transcripts for TLR-2, TLR-4 and TLR-5 (Fig. 6). In contrast to other disaggregated crypt epithelial cells, side population/putative stem cells adhere readily to monolayers of intestinal myofibroblasts 24. Such co-cultures were used to demonstrate immunoreactivity for not only BerEP4 (Fig. 7a), but also TLR-2 (Fig. 7b), TL-4 (Fig. 7c) and TLR-5 (Fig. 7d). In contrast to the epithelial cells, myofibroblast immunoreactivity for TLR-2, TLR-4 and TLR-5 in these co-cultures was weak.

### Studies in isolated intestinal myofibroblasts

**Fig 8:**
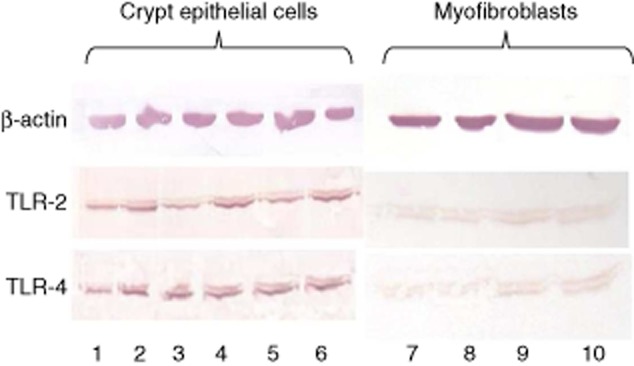
Toll-like receptor (TLR)-2 and TLR-4 protein expression in colonic crypt epithelial cells and myofibroblasts. Colonic crypt epithelial cells were isolated from histologically normal colonic (lanes 1 and 2) mucosal samples and those affected by active Crohn's colitis (lanes 3 and 4) and active ulcerative colitis (lanes 5 and 6). Primary human colonic myofibroblasts were isolated from histologically normal colonic mucosal samples (lanes 7–10). Cell lysates were used for Western blot analysis. For crypt epithelial cell lysates, 20 μg of total protein was applied per lane. Although higher amounts of myofibroblast cell lysates (40 μg in each of lanes 7 and 8; 80 μg in each of lanes 9 and 10) were used, bands for TLR-2 and TLR-4 were stronger in lysates of crypt epithelial cells (lanes 1–6).

Using conventional RT–PCR, myofibroblasts isolated from normal control and active IBD mucosal samples showed PCR products specific for TLR-2 and TLR-4 (not shown). TLR-2 and TLR-4 protein expression was confirmed by Western blot analysis, but the level of expression was much lower than that for isolated crypt epithelial cells (Fig. 8).

## Discussion

To date, the role of TLRs in intestinal epithelial cells has been investigated predominantly in mice and human cell lines, with only limited studies in primary human mucosal epithelial cells. Heterogeneity in expression of TLR-4 has been reported in epithelial cell lines 28–30. In tissue sections of human intestinal mucosal samples, reports of epithelial expression of TLR-2 and TLR-4 have been inconsistent 13–16.

In findings that we believe have not been reported previously, our studies using isolated and disaggregated colonic crypt epithelial cells consistently showed expression of not only transcripts, but also TLR-2 and TLR-4 protein on the cell surface. Compared to histologically normal controls, crypt epithelial cells isolated from colonic mucosal samples affected by ulcerative colitis and Crohn's disease demonstrated enhanced expression of TLR-2 and TLR-4 transcripts and cell surface protein. These studies suggest greater capacity for colonic crypt epithelial cells in inflammatory bowel disease to respond to luminal microbial products that bind these receptors.

The enhanced epithelial expression of TLR-2 and TLR-4 is likely to have occurred in response to proinflammatory cytokines 28,31. However, we report for the first time that expression of transcripts for both TLR-2 and TLR-4 was similar in crypt epithelial cells isolated from histologically normal and inflamed parts of colectomy specimens with left-sided ulcerative colitis. Indeed, compared to control normal colonic mucosal samples, there was enhanced expression of TLR-4 in crypt epithelial cells isolated from histologically normal proximal colon of these colectomy specimens with distal ulcerative colitis. It is possible, therefore, that compared to those without IBD, there is enhanced constitutive expression of TLR-4 in crypt epithelial cells throughout the colon of patients with ulcerative colitis.

Limitations of our studies include the use of relatively small numbers of samples, which were obtained from operation resection specimens. Although the control histologically normal mucosal samples were obtained distant from the cancer in the resection specimen, it is conceivable that the presence of the neoplasm may affect TLR expression in the adjacent tissue. We believe this is unlikely, but future studies using samples from patients without cancer can address this issue. Studies using epithelial cells isolated from endoscopic biopsies from IBD patients while not on any treatment will also be of interest.

Isolated and disaggregated crypt epithelial cells used in our studies also contain stem cells, which give rise to the progeny that differentiate as they migrate to the surface of the mucosa.

Stem cells with so-called side population characteristics (based on the ability to efflux the DNA-binding dye Hoechst 33342) have been characterized in the bone marrow 26 and murine intestine 32,33. We have shown previously that isolated and purified (using cell sorter) putative human colonic epithelial stem cells with side population characteristics adhere to monolayers of primary human colonic myofibroblasts 24. In novel studies, we now report that these putative human colonic stem cells express TLR-2, TLR-4 and TLR-5. Wnt signalling is important in regulating stem cell function and a recent study has reported the ability of TLR-4 to activate the canonical Wnt pathway in colonic epithelial cell lines 34. Expression of TLR-4 in Lgr5-positive murine small intestinal stem cells has also been reported 35. Moreover, loss of TLR-4 in murine intestinal epithelial cells has been shown to lead to goblet cell differentiation, probably via suppression of Notch signalling in stem cells 36.

Studies suggest that, in contrast to the epithelium, TLR signalling in lamina propria cells leads to proinflammatory responses 37. Beneficial effects mediated by TLR-2 and TLR-4 receptors in the intestinal epithelium have been observed predominantly in models of radiation injury 11 and colitis induced by dextran sulphate sodium 9,38,39 and *Citrobacter rodentium* 40. Stem cells are sensitive to radiation 3, and our studies suggest that the protective effects of TLR-4 11 and TLR-5 10 ligands could be mediated directly via receptors expressed on the surface of these cells.

Human intestinal myofibroblasts, which demonstrate characteristics of fibroblasts and smooth muscle cells 25, are located immediately subjacent to the epithelium. In the crypt, they represent an important component of the stem cell niche 19,20, and have also been implicated in adenoma initiation and growth 41. The demonstration of intestinal myofibroblast expression of TLRs 21,22 represents an increasing appreciation of their role in mediating mucosal immunological responses 42,43. Our studies have confirmed the expression of TLR-2 and TLR-4 in myofiboblasts isolated from normal colonic mucosal samples, and report for the first time that levels of the proteins were lower than in isolated crypt epithelial cells. Future studies can investigate the significance of the differences between crypt epithelial cells and myofibroblasts in expression of these TLRs.

## Supporting Information

**Fig. S1.** Toll-like receptor (TLR)-2, TLR-4 and hypoxanthine–guanine phosphoribosyltransferase (HPRT) mRNA expression by isolated and disaggregated intestinal crypt epithelial cells obtained from histologically normal (control) small intestine (lane 1), inflamed small bowel Crohn's disease (lane 2), histologically normal (control) large intestine (lane 3), inflamed colonic Crohn's disease (lane 4) and inflamed large intestinal mucosal samples affected by ulcerative colitis (lane 5). Following reverse transcription, extracted RNA was used for polymerase chain reaction (PCR) using specific primer pairs and controls included omission of reverse transcriptase (lane 6) and lack of cDNA template (lane 7). The figure is representative of experiments undertaken using crypt epithelial cells isolated from ≥ 5 specimens for each group identified in lanes 1–5. L = DNA size markers. **Table S1.** Details of patients studied. TNFα = tumour necrosis factor-α. **P* < 0·05; ***P* < 0·01 *versus* healthy controls. **Table S2.** Relative quantitative expression of Toll-like receptor (TLR)-2 and TLR-4 mRNA transcripts in isolated and disaggregated colonic and small intestinal crypt epithelial cells obtained from histologically normal control mucosal samples and those affected by active ulcerative colitis (UC), Crohn's colitis and ileal Crohn's disease. Extracted RNA was used for real-time reverse transcription–polymerase chain reaction (RT–PCR) and data for UC and Crohn's disease are presented as ‘fold change’ in expression of transcripts compared to mean expression in the control group in which the crypt epithelial cells were obtained from histologically normal colonic and small intestinal mucosal samples. IQR = interquartile range. **Table S3.** Quantitative surface Toll-like receptor (TLR)-2 and TLR-4 protein expression by colonic crypt epithelial cells. Isolated and disaggregated crypt epithelial cells were obtained from mucosal samples affected by active Crohn's colitis, active ulcerative colitis or from histologically normal control colonic tissue. The cells were labelled with anti-BerEP4-fluorescein isothiocyanate (FITC) antibody and either anti-TLR-2 allophycocyanin (APC), anti-TLR-4-APC or isotype control monoclonal antibodies and analysed by flow cytometry. Surface TLR-2 and TLR-4 protein-associated median fluorescence intensity was determined in BerEP4-positive (gated) epithelial cells. IQR = interquartile range.
