# Supplementary material for: Toll-like receptor expression in crypt epithelial cells, putative stem cells and intestinal myofibroblasts isolated from controls and patients with inflammatory bowel disease
Source: Clin Exp Immunol. 2014 Sep 4;178(1):28–39. doi: 10.1111/cei.12381 (PMC4231243; doi:10.1111/cei.12381)
Supplement: Supplementary file 1 — Fig. S1. Toll-like receptor (TLR)-2, TLR-4 and hypoxanthine–guanine phosphoribosyltransferase (HPRT) mRNA expression by isolated and disaggregated intestinal crypt epithelial cells obtained from histologically normal (control) small intestine (lane 1), inflamed small bowel Crohn's disease (lane 2), histologically normal (control) large intestine (lane 3), inflamed colonic Crohn's disease (lane 4) and inflamed large intestinal mucosal samples affected by ulcerative colitis (lane 5). Following reverse transcription, extracted RNA was used for polymerase chain reaction (PCR) using specific primer pairs and controls included omission of reverse transcriptase (lane 6) and lack of cDNA template (lane 7). The figure is representative of experiments undertaken using crypt epithelial cells isolated from ≥ 5 specimens for each group identified in lanes 1–5. L = DNA size markers. Table S1. Details of patients studied. TNFα = tumour necrosis factor-α. *P < 0·05; **P < 0·01 versus healthy controls. Table S2. Relative quantitative expression of Toll-like receptor (TLR)-2 and TLR-4 mRNA transcripts in isolated and disaggregated colonic and small intestinal crypt epithelial cells obtained from histologically normal control mucosal samples and those affected by active ulcerative colitis (UC), Crohn's colitis and ileal Crohn's disease. Extracted RNA was used for real-time reverse transcription–polymerase chain reaction (RT–PCR) and data for UC and Crohn's disease are presented as ‘fold change’ in expression of transcripts compared to mean expression in the control group in which the crypt epithelial cells were obtained from histologically normal colonic and small intestinal mucosal samples. IQR = interquartile range. Table S3. Quantitative surface Toll-like receptor (TLR)-2 and TLR-4 protein expression by colonic crypt epithelial cells. Isolated and disaggregated crypt epithelial cells were obtained from mucosal samples affected by active Crohn's colitis, active ulcerative colitis or [file cei0178-0028-sd1.zip › Supplementary information.docx]

Supplementary information to the manuscript:

**Toll-like receptor expression in crypt epithelial cells, putative stem cells and intestinal myofibroblasts isolated from controls and patients with inflammatory bowel disease.**

M Brown, K R Hughes, S Moossavi, A Robins, Y R Mahida.

TLR2, TLR4 and HPRT mRNA expression by isolated and disaggregated intestinal crypt epithelial cells obtained from histologically normal (control) small intestine (lane 1), inflamed small bowel Crohn’s disease (lane 2), histologically normal (control) large intestine (lane 3), inflamed colonic Crohn’s disease (lane 4) and inflamed large intestinal mucosal samples affected by ulcerative colitis (lane 5). Following reverse transcription, extracted RNA was used for PCR using specific primer pairs and controls included omission of reverse transcriptase (lane 6) and lack of cDNA template (lane 7). The figure is representative of experiments undertaken using crypt epithelial cells isolated from >5 specimens for each group identified in lanes 1 - 5. L – DNA size markers.

**Supplementary Table 1.**

|  | Large intestine | | | Small intestine | |
| --- | --- | --- | --- | --- | --- |
|  | Normal  control | Ulcerative colitis | Crohn’s colitis | Normal control | Crohn’s ileitis |
| Number | 11 | 18 | 11 | 7 | 7 |
| Age: mean (SEM) | 70.5 (4.1) | 51.5 (4.7)^**^ | 48.9 (4.1)^**^ | 74.1 (3.4) | 44.3 (7.2)^**^ |
| Male / female (% female) | 7/4 (64) | 9/9 (50) | 2/9 (18) | 5/2 (71) | 2/5 (29) |
| Medications |  |  |  |  |  |
| - Mesalazine | 0 | 11^**^ | 3 | 0 | 0 |
| - Thiopurine | 0 | 8^*^ | 3 | 0 | 4 |
| - Corticosteroid | 0 | 3 | 4 | 0 | 1 |
| - Methotrexate | 0 | 2 | 2 | 0 | 0 |
| - Anti-TNFα | 0 | 0 | 6^*^ | 0 | 2 |
| - Ciclosporin | 0 | 1 | 0 | 0 | 0 |
| - Metronidazole | 0 | 1 | 0 | 0 | 0 |

Details of patients studied. TNFα, tumour necrosis factor-α. *p<0.05; **p<0.01 versus healthy controls.

**Supplementary Table 2.**

| Sample | Sample group | Number | Relative TLR2 mRNA expression | | Relative TLR4 mRNA expression | |
| --- | --- | --- | --- | --- | --- | --- |
|  |  |  | Median  (IQR) | p value | Median (IQR) | p value |
| Colonic crypt epithelial cells | Normal control | 11 | 1.0  (0.59 – 1.42) | - | 1.0  (0.82 – 1.37) | - |
|  | Ulcerative colitis | 13 | 3.18  (1.03 – 10.4) | 0.003 | 2.33  (1.15 – 4.45) | 0.024 |
|  | Crohn’s colitis | 11 | 3.45  (0.80 – 5.40) | 0.012 | 1.71  (1.0 – 4.32) | 0.042 |
| Small intestinal crypt epithelial cells | Normal control | 7 | 1.0  (0.77 – 1.22) | - | 1.0  (0.72 – 1.89) | - |
|  | Crohn’s ileitis | 7 | 1.72  (0.52 – 2.74) | 0.208 | 1.84  (1.43 – 4.66) | 0.030 |

Relative quantitative expression of TLR2 and TLR4 mRNA transcripts in isolated and disaggregated colonic and small intestinal crypt epithelial cells obtained from histologically normal control mucosal samples and those affected by active ulcerative colitis, Crohn’s colitis and ileal Crohn’s disease. Extracted RNA was used for real time RT-PCR and data for UC and Crohn’s disease are presented as “fold change” in expression of transcripts compared to mean expression in the control group in which the crypt epithelial cells were obtained from histologically normal colonic and small intestinal mucosal samples. IQR: inter-quartile range.

**Supplementary Table 3.**

| Sample | Sample group | Number | TLR2 protein associated median fluorescence intensity | | TLR4 protein associated median fluorescence intensity | |
| --- | --- | --- | --- | --- | --- | --- |
|  |  |  | Median  (IQR) | p value | Median (IQR) | p value |
| Colonic crypt epithelial cells | Normal control | 7 | 10.1  (2.4 – 29.0) | - | 12.1  (4.9 – 25.3) | - |
|  | Ulcerative colitis | 4 | 89.1  (39.0 – 146.1) | 0.006 | 73.0  (31.1 – 179.4) | 0.024 |
|  | Crohn’s colitis | 8 | 65.8  (19.3 – 126.3) | 0.029 | 69.7  (26.4 – 115.1) | 0.021 |

Quantitative surface TLR2 and TLR4 protein expression by colonic crypt epithelial cells. Isolated and disaggregated crypt epithelial cells were obtained from mucosal samples affected by active Crohn’s colitis, active ulcerative colitis or from histologically normal control colonic tissue. The cells were labelled with anti-BerEP4-FITC antibody and either anti-TLR2-APC, anti-TLR4-APC or isotype control monoclonal antibodies and analysed by flow cytometry. Surface TLR2 and TLR4 protein associated median fluorescence intensity was determined BerEP4-positive (gated) epithelial cells. IQR: inter-quartile range.
